# Supplementary material for: Inactive S. aureus Cas9 downregulates alpha-synuclein and reduces mtDNA damage and oxidative stress levels in human stem cell model of Parkinson’s disease
Source: Sci Rep. 2023 Oct 18;13:17796. doi: 10.1038/s41598-023-45078-3 (PMC10584834; doi:10.1038/s41598-023-45078-3)
Supplement: Supplementary file 1 — Supplementary Information. [file 41598_2023_45078_MOESM1_ESM.pdf]

Supplementary Data

Inactive S. aureus Cas9 downregulates alpha-synuclein and reduces mtDNA damage and oxidative stress levels in human stem cell model of Parkinson's disease

Danuta Sastre[1^+], Faria Zafar[1+], C. Alejandra Morato Torres[1], Desiree Piper[2], Deniz Kirik[4], Laurie H. Sanders[5], L. Stanley Qi[3], Birgitt Schüle[1*]

[1] Stanford University School of Medicine, Department of Pathology, Stanford, CA 94305, U.S.A.

[2] San Jose State University, Department of Biological Sciences, San Jose, 95192 CA, U.S.A.

[3] Stanford University, Department of Bioengineering, Stanford, CA 94305, U.S.A.

[4] Department of Experimental Medical Science, Lund University, Lund, Sweden

[5] Departments of Neurology and Pathology, Duke Center for Neurodegeneration and Neurotherapeutics, Duke University Medical Center, Durham, NC 27710, U.S.A.

*corresponding author

+these authors contributed equally to this work

^ Current affiliation: SRI International, Biosciences Division, Menlo Park, 94025 CA, U.S.A

*** Corresponding author**

Birgitt Schüle, MD, Dr. med.

Stanford University School of Medicine

Department of Pathology

300 Pasteur Dr., R271/217

Stanford, CA 94305

E-mail address: bschuele@stanford.edu

Supplementary Table 1: Genomic position of sgRNA and transcription start sites in the promoter region of *SNCA* gene (GRCh37/hg19).

sgRNA/TSS	Position (GRCh37/hg19)
155F	chr4: 90756784-90756804
155R	chr4: 90757811-90757831
178R	chr4: 90757834-90757854
202F	chr4: 90757831-90757851
228R	chr4: 90757884-90757904
267R	chr4: 90757923-90757943
317R	chr4: 90757973-90757993
382R	chr4: 90758038-90758058
417F	chr4: 90757073-90757093
438F	chr4: 90758067-90758087
453R	chr4: 90758109-90758129
469F	chr4: 90758098-90758118
479R	chr4: 90758135-90758155
510F	chr4: 90757139-90757159
532R	chr4: 90759188-90759208
539F	chr4: 90757168-90757188
552R	chr4: 90757208-90757228
564R	chr4: 90759220-90759240
571F	chr4: 90758200-90758220
626R	chr4: 90757282-90757302
629F	chr4: 90757258-90757278
645F	chr4: 90759274-90759294
681R	chr4: 90757337-90757357
696F	chr4: 90757352-90757372
716R	chr4: 90757372-90757392
738R	chr4: 90757394-90757414
792F	chr4: 90759421-90759441
802F	chr4: 90758431-90758451
821F	chr4: 90758450-90758470
836F	chr4: 90758492-90758512
845F	chr4: 90759474-90759494
849R	chr4: 90759505-90759525
TSS1	chr4:90,759,446-90,759,448
TSS2.1	chr4:90,758,348-90,758,350;
TSS2.2	chr4:90,758,123-90,758,125
TSS3	chr4:90,757,362-90,757,364.

TSS = transcription start site

Supplementary Table 2: Off-target analysis summary for sgRNAs functionally tested in SNCA-triplication iPSCs

TSS	ID	Genomic Position (GRCh37/hg19)	Off-target analysis per number of mismatches				*SNCA* mRNA downregulation in *SNCA*-triplication iPSC
			1	2	3	4	
1	792F	chr4: 90759421-90759441	-	-	-	16 (2 exonic, 5 intronic, 9 intergenic)	25%
2.2	382R	chr4: 90758038-90758058	-	2 (1 intronic, 1 intergenic)	2 (1 intronic, 1 intergenic)	15 (8 intronic, 7 intergenic)	75%
2.2	228R	chr4: 90757884-90757904	-	1 (intergenic)	1 (exonic)	7 (1 exonic, 1 intronic, 5 intergenic)	50%
3	510F	chr4: 90757139-90757159	-	-	-	6 (2 intronic, 4 intergenic)	75%

Supplementary Table 3: SYBR™ green primers for the detection of SNCA gene isoforms for qPCR.

Target	Source	Primer	Primer Sequence 5'-3'	Product size	Tm °C (Avg)	Tm °C (Beacon)	Target	Tm °C
*SNCA*140	McLean et al.[1]	Fwd	AAAACCAAGGAGGGAGTGGT	238	55.3	55.62	exon 3	55
		Rev	TGTCAGGATCCACAGGCATA			55.01	exon 5	
*SNCA*126	Bungeroth et al.[2]	Fwd	AAAGAGGGTGTTCTCTATGTAGTGG	185	56.31	57.84	exon 2-4	55
		Rev	TGTGGGGCTCCTTCTTCAT			54.78	exon 5	
*SNCA*112	Bungeroth et al.[2]	Fwd	TGTCAGGATCCACAGGCATA	178	55.7	55.88	exon 3	55
		Rev	ATACCCTTCCTTGCCCAACT			55.46	exon 4-6	
*SNCA*98	Piper	Fwd	CTCTATGTAGTGGCTGAGAAGA	165	54.7	53.92	exon 2-4	55
		Rev	TGTCAGGATCCACAGGCATA			55.48	exon 4-6	
GAPDH	Piper	Fwd	CATCACCATCTTCCAGGAGC	182	56.2	55.01		55
		Rev	ATGACGAACATGGGGGCATC			57.7		

Tm, melting temperature; Avg, average.

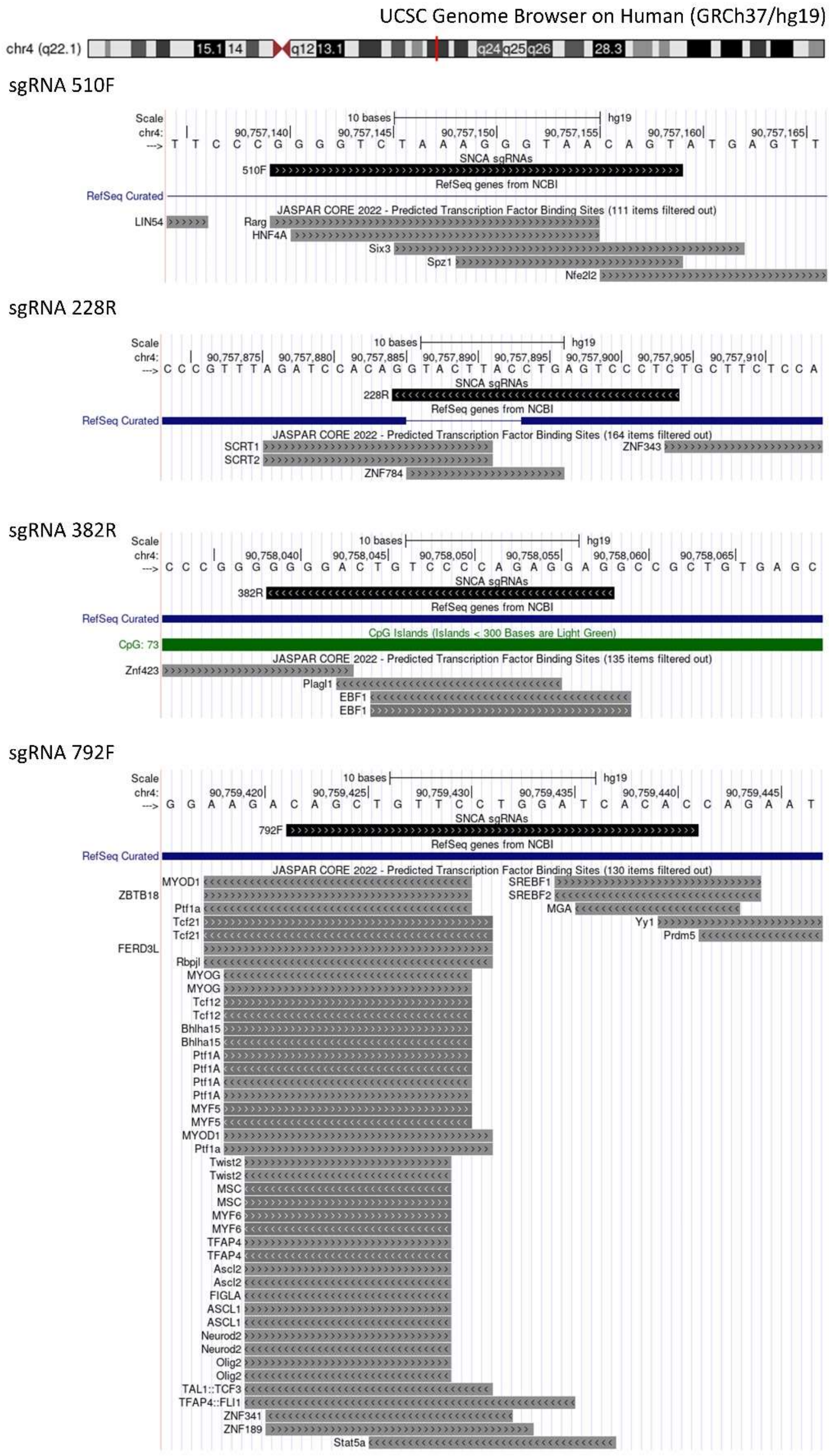

Supplementary Figure 1. Predicted transcription factor binding sites for four sgRNAs tested *in vitro* in UCSC Genome Browser with JASPAR database.

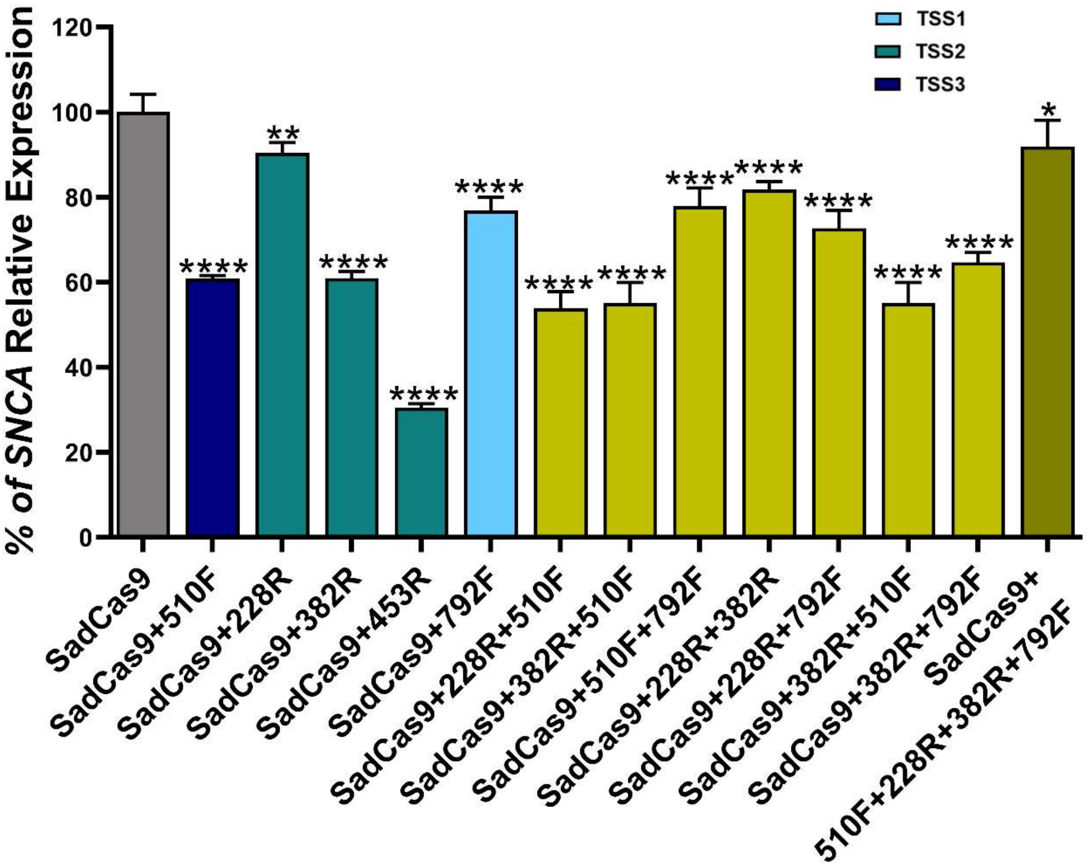

Supplementary Figure 2. Testing *SNCA* sgRNAs combinations in HEK293 cells. *SNCA* mRNA expression in HEK293 cells transiently transfected with SadCas9 and individual sgRNAs or combination of sgRNAs from all three TSSs. Relative expression of *SNCA* mRNA was measured by qPCR and normalized to the expression of GAPDH. Calibrator sample is transfected with only SadCas9 without any sgRNA (grey). Data are displayed as mean ± SD in triplicates from one experiment. Differences between groups were detected by ANOVA with Dunnet post-test (p<0.1*, p<0.01**, p<0.001***, and p<0.0001****)

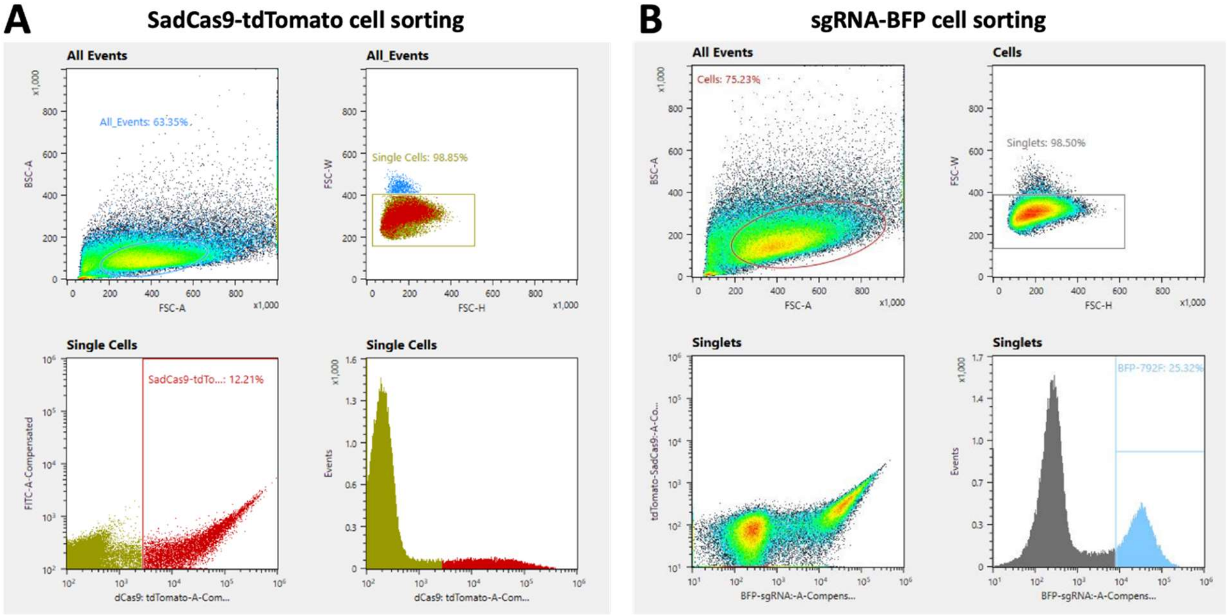

Supplementary Figure 3: Fluorescence-activated cell sorting of SadCas9 expression in human iPSCs. (**A**) FACS isolation of *SNCA*-triplication for SadCas9-tdTomato and (**B**) for sgRNA-BFP. iPSCs were transduced with 12.5 µL of concentrated lentivirus SadCas9-tdTomato and 12.5 µL of concentrated lentivirus rtTA in 400 µL of StemFlex medium with 6 µg/mL of polybrene. After 24 h, media was changed, and cells were cultured until they reached 100% confluency. Confluent cell cultures were expanded into 6-well plates for 1-2 weeks. To induce SadCas9 expression, cells were treated with 1 µg/mL doxycycline for 24 h prior to cell sorting. Cells were sorted based on expression of tdTomato as a marker for expression of SadCas9. Sorted cells were a heterogeneous population showing different levels of SadCas9 expression. These cells were expanded for another 1-2 weeks. To normalize the level of SadCas9 expression and allow accurate comparison between sgRNAs, SadCas9-iPSCs were clonally selected using serial dilution method. A final dilution of 0.5 cells/100 µL of medium was plated into 96-well plates. sgRNA infection of clonal SadCas9-iPSCs was performed using 25 µL of concentrated lentivirus sgRNA in 400 µL of StemFlex medium with 6 µg/mL of polybrene. Cells were expanded into 6-well plates for 1-2 weeks prior to sorting.

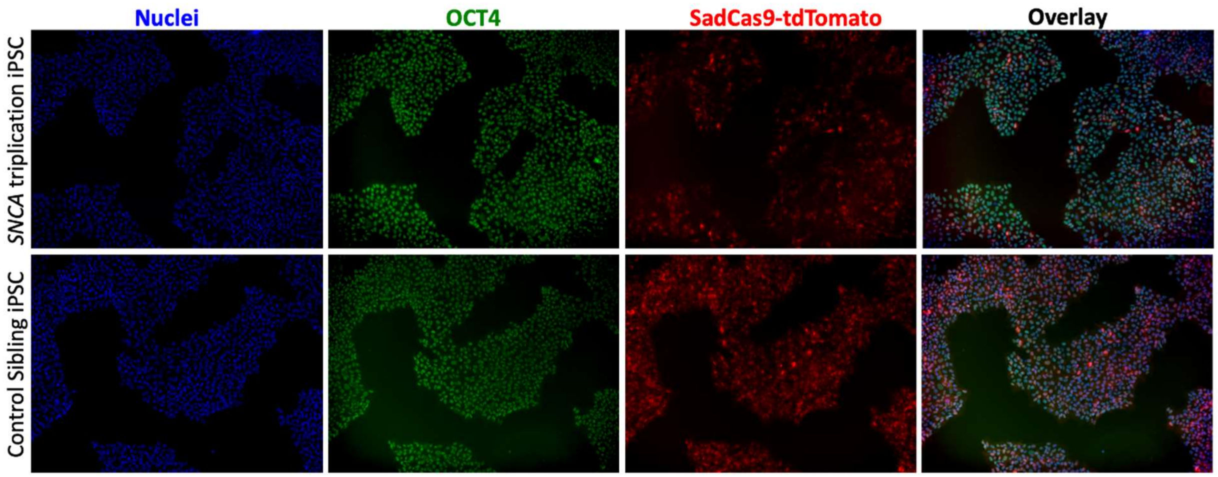

Supplementary Figure 4: Patient derived iPSCs remain pluripotent after lentiviral integration of SadCas9 expression cassette. Representative 10X images of *SNCA*-triplication iPSCs (upper panel), and control sibling iPSCs (lower panel) expressing pluripotency marker OCT4 (green) while expressing SadCas9-tdTomato (red) at 24 h post-treatment with 1 µg/mL doxycycline.

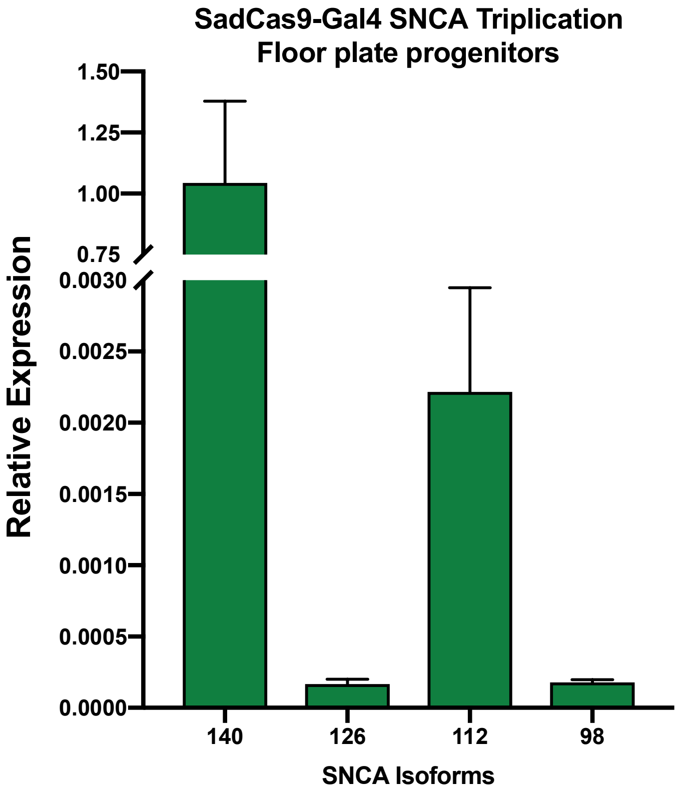

Supplementary Figure 5. Relative expression of *SNCA* isoforms in floor-progenitor cells derived from *SNCA*-triplication iPSCs.

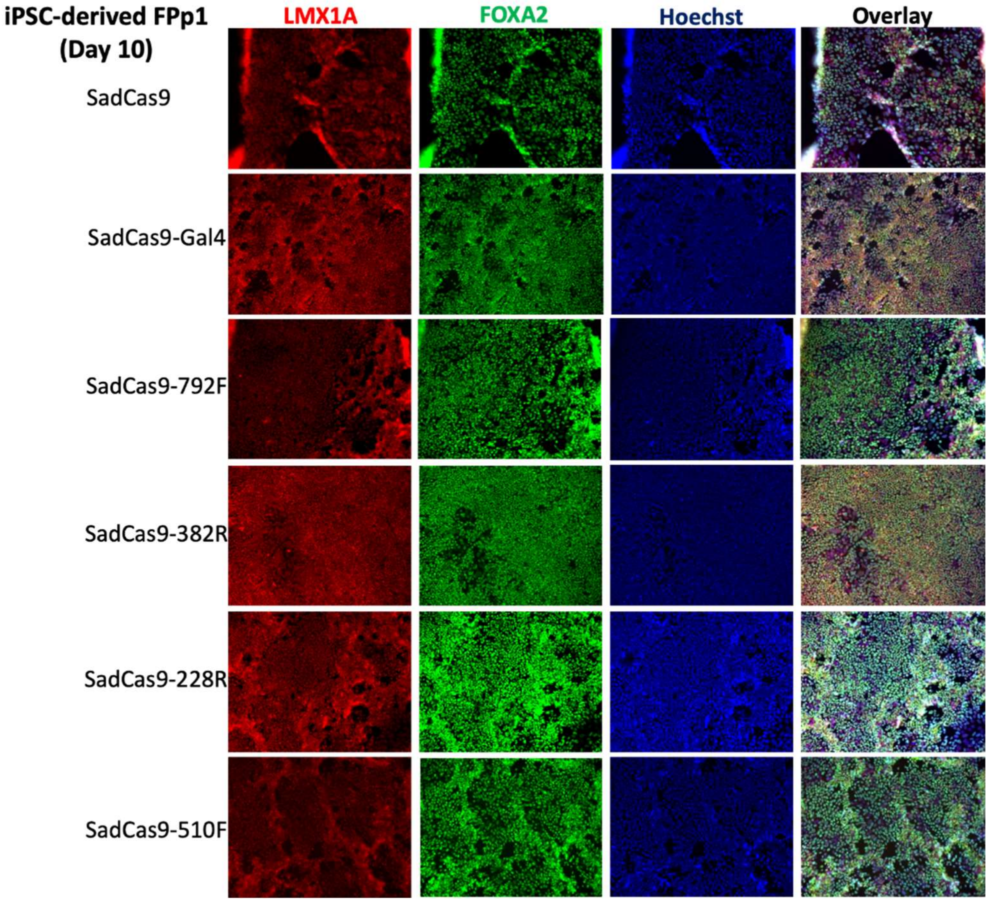

Supplementary Figure 6. Neuronally differentiated floorplate progenitors (FPp1 at day 10 of differentiation) derived from SadCas9/sgRNA expressing iPSCs. All lines express floorplate progenitor markers LMX1A and FOXA2.

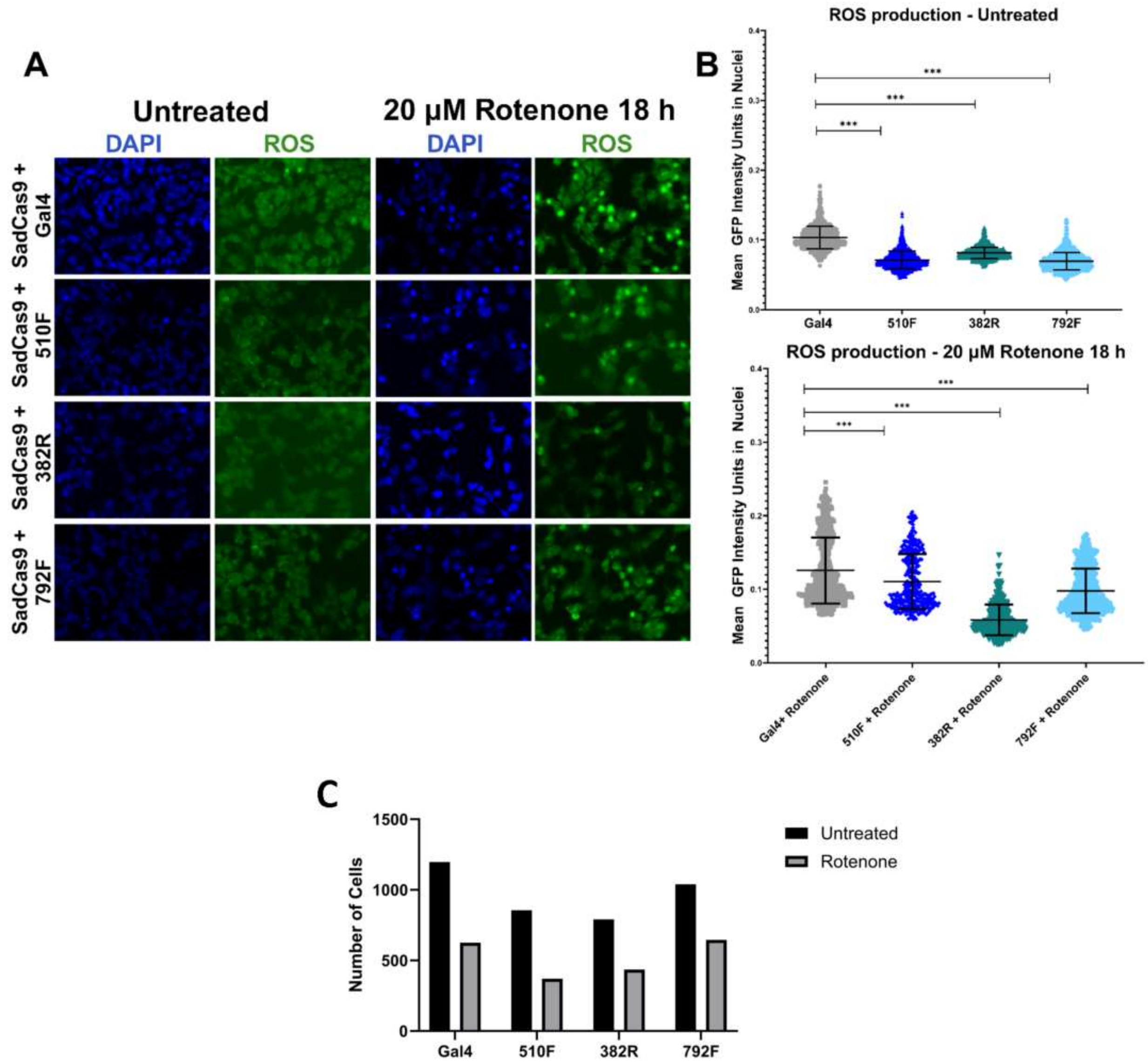

Supplementary Figure 7. CellROX® Green Oxidative Stress assay in iPSC-derived floorplate progenitors from patient with *SNCA* triplication. The figure illustrates measurement of reactive oxygen species (ROS) using a fluorogenic probe that presents with a strong fluorogenic signal upon oxidation and localizes to nuclei. (**A**) Representative images of reactive oxygen species (ROS), (**B**) Mean intensity of ROS per nuclei in rotenone treated cells. The upper panel represent the untreated naïve condition, and lower panels represent 18 h rotenone treatment (n=9 images per condition; 5,957 nuclei analyzed). Differences between groups were detected by ANOVA (p<0.001 ***), (**C**) Cell counts with and without 18 h rotenone treatment.

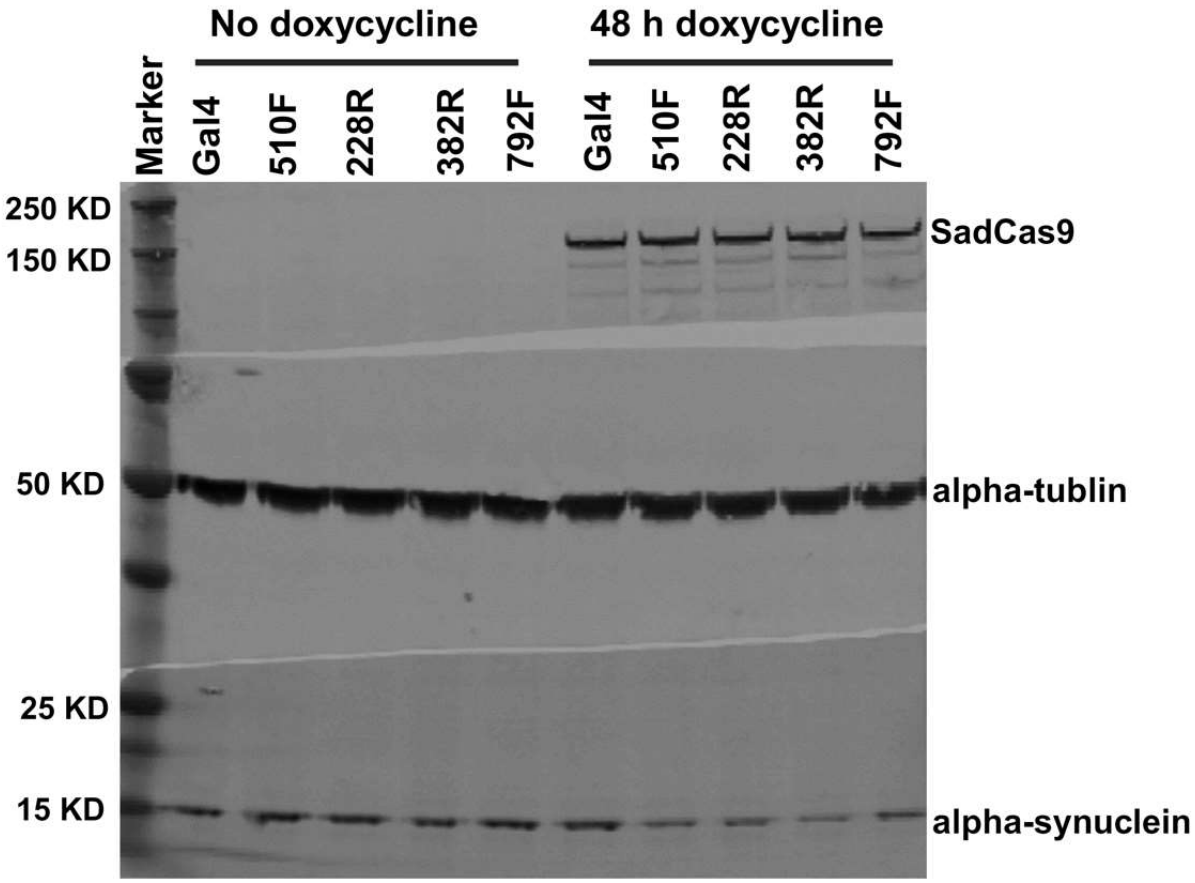

Supplementary Figure 8. Uncropped Western blot from Figure 2D. Western blot representation of total SadCas9 and total alpha-synuclein protein.

Supplementary Figure 9. Plasmid map and sequence of the SadCas9 and tdTomato reporter (Addgene #209298)

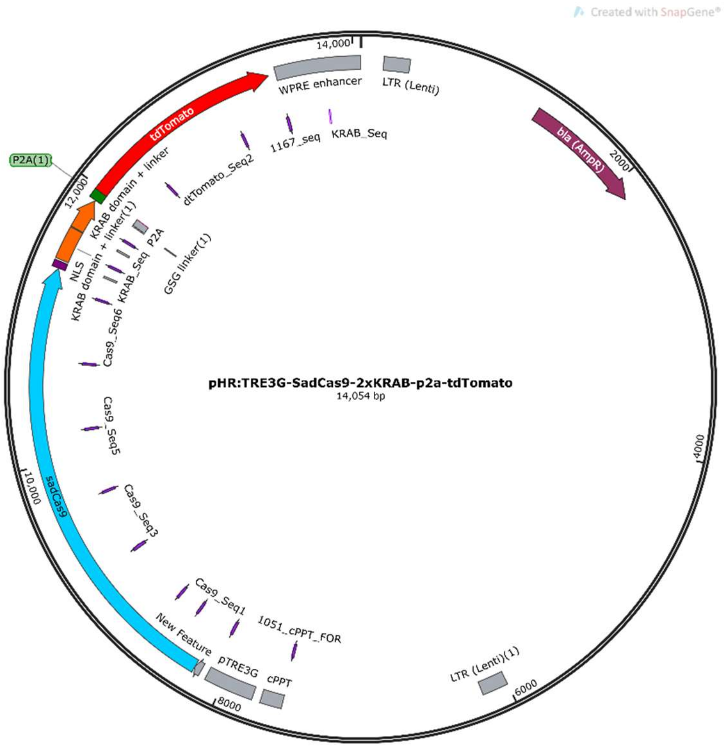

Supplementary methods:

CellROX® Green Oxidative Stress staining (Thermo Fisher, Cat. No. C10444): CellROX® Green Reagent is a DNA dye, thus strong nuclear signal indicates higher production of reactive oxygen species. We treated the cells with 20 µM rotenone for 18 hrs. Culture medium was removed and replaced with HBSS solution containing 5 µM of CellROX® Reagent and incubated for 30 min at 37°C. Cells were incubated at 37°C for 30 minutes, then fixed with 10% buffered neutral formalin for 15min. Hoechst was used as nuclear counterstain. Excitation and emission were 485/520nm (Green). Fluorescence was measured within 24 h after staining.

References

1 McLean, J. R., Hallett, P. J., Cooper, O., Stanley, M. & Isacson, O. Transcript expression levels of full-length alpha-synuclein and its three alternatively spliced variants in Parkinson's disease brain regions and in a transgenic mouse model of alpha-synuclein overexpression. *Molecular and cellular neurosciences* **49**, 230-239, doi:10.1016/j.mcn.2011.11.006 (2012).

2 Bungeroth, M. *et al.* Differential aggregation properties of alpha-synuclein isoforms. *Neurobiol Aging* **35**, 1913-1919, doi:10.1016/j.neurobiolaging.2014.02.009 (2014).
